# Interpreting Mutation Co-Occurrence in Cancer Genomics Under Biological Context

**DOI:** 10.3390/cancers18172833

**Published:** 2026-09-01

**Authors:** Yong Hun Jang, Woochang Hwang

**Affiliations:** 1 Department of Pediatrics, College of Medicine, Hanyang University, Seoul 04763, Republic of Korea; ryanjang93@hanyang.ac.kr; 2 Hanyang Institute of Bioscience and Biotechnology, Hanyang University, Seoul 04763, Republic of Korea; 3 Department of Biomedical Science, Graduate School of Biomedical Science and Engineering, Hanyang University, Seoul 04763, Republic of Korea; 4 Department of Pre-Medicine, College of Medicine, Hanyang University, Seoul 04763, Republic of Korea

**Keywords:** mutation co-occurrence, mutual exclusivity, clonal evolution, intratumor heterogeneity, single-cell sequencing

## Abstract

Somatic mutation patterns observed in cancer genomes are widely used to generate hypotheses about functional relationships among cancer genes and signaling pathways. However, mutation co-occurrence and mutual exclusivity are assessed at multiple levels, including cohorts, bulk specimens, lesions, regions, clones, and individual cells, although each observational level supports a different scope of inference. In this structured narrative review, we clarify these inferential boundaries and distinguish marginal from conditional association, as well as negative association from complete mutual exclusivity. A hypothetical numerical example of Simpson’s reversal illustrates how marginal and conditional associations can differ and why negative association with non-zero overlap should be distinguished from complete mutual exclusivity. We then synthesize evidence from bulk, multi-region, phylogenetic, and single-cell analyses to examine spatial and clonal localization, interclonal cooperation, single-cell error and detection power, and genetic versus non-genetic resistance. We also provide a decision guide for method selection and a staged framework for functional validation. Overall, statistical association, physical localization, and functional interaction are related but distinct inferential targets that require different data, assumptions, and forms of validation.

## Simple Summary

Cancer develops through genetic changes that allow cell populations to grow, survive, and evolve. In cancer genome studies, mutations are described as co-occurring when detected together, but this pattern does not prove biological cooperation. Associations may change when tumors with different clinical or molecular characteristics are pooled, and bulk specimens cannot show whether mutations occur in the same region, clone, or cell. This review examines mutation co-occurrence across clinical context, tumor evolution, spatial heterogeneity, and single-cell composition. A hypothetical numerical example shows how pooling tumors with different mutation prevalences can reverse the apparent direction of association. We also summarize approaches for statistical testing, clonal inference, single-cell interpretation, and functional validation. The review emphasizes that statistical association, physical localization, and functional interaction are distinct questions requiring different evidence.

## 1. Introduction

Cancer genomics provides essential biological insight for understanding the evolutionary process by which diverse cell populations within a malignant tumor compete with and adapt to one another. The core process of cancer initiation and progression occurs through the sequential accumulation of mutations that confer a selective advantage on cells. This process of cancer evolution is known to involve 12 major signaling pathways and 140 core genes that regulate cell fate, cell survival, and genome maintenance [1]. Furthermore, the expansion of clonal populations that have acquired a selective evolutionary advantage, together with intratumor heterogeneity (ITH), constitutes a key mechanism driving cancer evolution [2]. Within this evolutionary framework, the patterns of mutations observed across the tumor genome have emerged as important clues for elucidating the functional relationships between cancer-associated genes and signaling pathways.

Mutation co-occurrence and mutual exclusivity are widely studied patterns describing relationships among alterations in cancer. At cohort resolution, co-occurrence denotes a positive association across independent tumors under a specified null model, whereas mutual exclusivity denotes a deficit of double-positive tumors; negative association can include non-zero overlap. Complete mutual exclusivity is the descriptive condition in which no double-positive observational unit is present. These statistical patterns can motivate hypotheses about cooperation, functional redundancy, lineage restriction, or synthetic lethality, but none of these mechanisms can be inferred from the pattern alone [3,4,5,6,7]. When clinically or biologically heterogeneous strata are pooled, the marginal association can differ from the stratum-specific or covariate-adjusted association. This aggregation problem must be separated from the within-specimen mixture of regions, subclones, or cells.

Existing reviews have already established the principles of subclonal reconstruction, branched evolution, multi-region sequencing, and computational mutual exclusivity analysis [5,8,9,10]. Building on that foundation, this review addresses a distinct practical question: how to align a biological claim about mutation co-occurrence with the observation unit, statistical assumptions, and evidence required to support it. To address this question, we formalize the aggregation problem, illustrate its consequences with a hypothetical numerical example, provide practical guidance for matching analytical methods to inferential questions, and outline a staged framework for functional validation. Accordingly, this review examines mutation co-occurrence across three levels of biological resolution. At the cohort level, associations must be interpreted in light of variation in tumor mutational burden (TMB), molecular subtype, and cohort composition. At the clone level, within-tumor co-detection should be interpreted in the context of clonal architecture, subclonal branching, spatial distribution, and phylogenetic lineage. At the single-cell level, data can assess whether co-detected mutations are observed in the same measured cell, segregated across distinct cell populations, or confined to a rare cellular population, subject to callability, error, and sampling limitations. Taken together, these levels define a cohort-to-clone-to-cell framework while distinguishing three connected inferential questions: statistical dependence across observational units, physical localization within a patient or tumor, and functional interaction within or between cell populations.

We conducted a structured narrative review. PubMed/MEDLINE and Europe PMC were searched from database inception through 3 August 2026. Searches combined cancer or neoplasm terms with mutation co-occurrence, mutual exclusivity, genetic interaction, epistasis, confounding, molecular subtype, ITH, variant allele frequency (VAF), cancer cell fraction (CCF), phylogeny, multi-region sequencing, single-cell DNA sequencing (scDNA-seq), interclonal cooperation, circulating tumor DNA, drug-tolerant states, and acquired resistance. Exact database-specific queries and the records returned on the search date are reproduced in Appendix A (Table A1). Preprints were included when they provided relevant methodological developments but were not used as principal evidence for definitive biological conclusions. Eligible articles introduced or benchmarked a relevant method, analyzed human cancer genomic data, established a measurement limitation, or experimentally tested a mechanistic interpretation. Because the search was designed for critical narrative synthesis rather than exhaustive enumeration, studies were purposefully selected according to direct relevance to the review’s inferential framework, methodological rigor, evidentiary level, and ability to illustrate a distinct analytical or biological limitation; priority was given to original method papers, benchmark or validation studies, and human studies with explicit observation units and uncertainty reporting. As a structured narrative review, the process did not include exhaustive retrieval, duplicate screening, or procedures compliant with the Preferred Reporting Items for Systematic Reviews and Meta-Analyses.

## 2. Conceptual Boundaries of Cohort-Level Mutational Patterns

**Figure 1:**
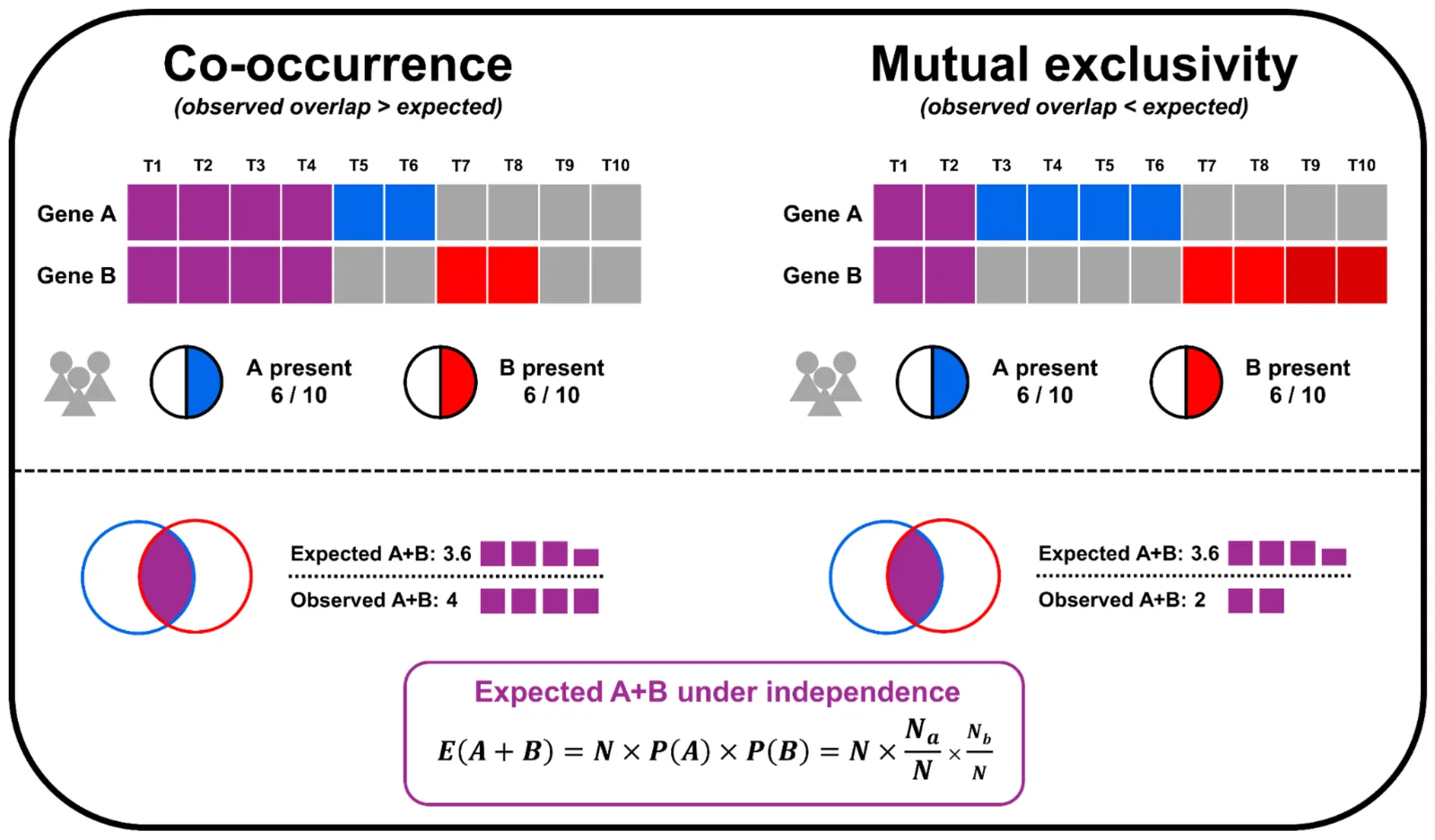
Schematic comparison of mutation co-occurrence and mutual exclusivity at the cohort level. Co-occurrence is illustrated by an observed A + B overlap greater than expected under independence, whereas mutual exclusivity is illustrated by an observed overlap lower than expected. Purple indicates A + B, blue A only, red B only, and gray neither mutation. Expected overlap is based on the marginal frequencies of mutations A and B, T, tumor.

The binary tumor–gene matrix is the standard representation for describing mutation patterns at the cohort level. In this matrix, each row represents an individual tumor sample, and each column represents a particular gene, indicating in binary form whether a mutation in that gene was detected in that sample. Statistical tests are then used to evaluate whether the observed frequency with which two or more mutations appear together differs significantly from the expected frequency under the hypothesis that each mutation occurs independently, thereby distinguishing co-occurrence from mutual exclusivity (Figure 1). Although this analytical approach is useful for the exploratory identification of relationships among mutations in large cohorts, it has fundamental limitations for interpreting the biological context of these patterns.

### 2.1. Observation Units and Evidence Boundaries

A cohort is a collection of observational units; a patient can contribute several lesions, specimens, or time points. A lesion is an anatomically distinguishable tumor deposit, a region is a sampled spatial subdivision of a lesion, a clone is an inferred lineage-defined population, and a cell is an individual physical unit. A specimen is a sampling unit that can contain more than one region or clone. In bulk sequencing, the detection of both alterations indicates that they are present in the analyzed material but does not establish that they reside within the same cell. Table 1 defines the supported inferences and principal limitations at the cohort, patient, lesion, region, clone, cell, and functional-system levels.

One limitation is that large-scale integrated cohort analyses generally include a mixture of primary samples, metastatic samples, and samples irrespective of treatment history. As a result, the observed mutation patterns may partly reflect cohort-level confounding when samples with different clinical and biological backgrounds are combined within a single tumor–gene matrix. This concern becomes especially relevant when tumors with different molecular subtypes are included in the same analytic population. For example, a study searching for mutually exclusive mutation combinations in breast cancer showed that *CDH1*, *AKT1*, and *PIK3CA* mutations are associated with the Luminal A subtype, and the mutation patterns observed at the cohort level are closely related to the distribution of the underlying molecular subtypes [11]. Breast cancer is composed of heterogeneous subtypes—such as Luminal A, Luminal B, HER2-positive, and basal-like—that have different driver mutation profiles [12]. When these heterogeneous molecular subtypes are merged and analyzed as a single cohort, the different mutation distributions of each subtype become intermingled within one tumor–gene matrix; consequently, the mutation patterns observed across the entire cohort may partly reflect the different alteration prevalences of the included subtypes, and it is therefore difficult to interpret them as biological evidence of direct functional cooperation or mutual exclusivity between two mutations (Table 1). This subtype mixture can attenuate, amplify, or reverse a marginal association, but a direction reversal must be demonstrated rather than assumed.

### 2.2. Formalizing Cohort Aggregation

Let A and B be binary alteration indicators for independent tumors and Z a clinical or molecular stratum such as subtype, histology, treatment status, or assay panel. The pooled, or marginal, association compares A and B without conditioning on Z; the conditional association compares them within strata of Z or in a covariate-adjusted model. Their covariance decomposes into within-stratum and between-stratum prevalence components:

(1) Cov(A,B)=E{Cov(A,B∨Z)}+Cov{E(A∨Z),E(B∨Z)}

On the covariance scale, a reversal from negative within-stratum association to positive marginal association can occur when the positive between-stratum prevalence component outweighs the average negative within-stratum covariance.

A hypothetical numerical example illustrates Simpson’s reversal. In a high-prevalence stratum, the A + B/A-only/B-only/neither counts are 61/19/19/1; in a low-prevalence stratum, they are 1/19/19/61. Each stratum has an odds ratio (OR) of 0.17, whereas the pooled counts 62/38/38/62 yield an OR of 2.66. The example contains non-zero overlap and therefore represents negative association, not complete mutual exclusivity. If P(A∩B∨Z=z)=0 in every exhaustive stratum at the same observational unit, pooled overlap also remains zero. Clinical-stratum aggregation and cellular mixture are distinct data-generating mechanisms. The former requires stratification, covariate adjustment, interaction assessment, and an explicit estimand. The latter requires spatial, clonal, or single-cell localization. A crude-to-adjusted OR change should be interpreted with care because ORs are non-collapsible and may differ even in the absence of confounding. Effect sizes, confidence intervals (CIs), stratum-specific estimates, and false discovery rate (FDR) control should also be reported.

A second limitation is that differences in TMB between samples can distort the interpretation of mutation patterns. Samples with high TMB contain more alteration calls, increasing the probability that unrelated events, including passenger alterations, will be observed together by chance. A pan-cancer study of 12 major cancer types from The Cancer Genome Atlas (TCGA) documented that mutation frequency and patterns differ by cancer type and vary with tissue of origin, carcinogenic exposures, and DNA repair deficiency status, illustrating heterogeneity that an association model may need to address [13]. Consistent with this concern, van de Haar et al. showed that many mutual-exclusivity patterns in cancer genomes can be influenced by subtype and tumor mutation burden rather than reflecting direct pathway epistasis [7]. Several statistical frameworks have been developed to reduce false-positive mutual exclusivity signals that can arise in cancer types containing hypermutated samples, such as colorectal and endometrial cancers. For example, methods have been proposed that incorporate each sample’s intrinsic mutational burden weight into the statistical model to evaluate mutual exclusivity more rigorously [14,15]. Models that represent driver and passenger processes separately can also reduce spurious associations in genes with high passenger mutation rates [16].

### 2.3. Limits of Binary Cohort Matrices for Evolutionary Interpretation

A third limitation is that the conventional binary matrix representation fails to reflect the temporal sequence of mutations during tumor evolution, their clonal or subclonal status, their co-occurrence within the same cell lineage, or whether they originate from spatially distinct tumor regions. For example, early clonal and late subclonal mutations—which carry different biological meanings—are treated as identical mutations within a binary matrix. In fact, a pan-cancer study found that approximately 15% of mutations in genes related to major pathways, such as the PI3K–AKT–mTOR pathway, were subclonal [17], suggesting that a driver alteration may be inferred as clonal in the sampled material or as subclonal and restricted to a subset of cancer cells. To overcome the limitations of this conventional one-dimensional representation, statistical models have been developed that jointly consider the temporal order of mutations and the evolutionary phylogeny of the tumor. By jointly inferring mutually exclusive oncogenic pathways and the temporal order in which mutations on those pathways arise, studies have been conducted that interpret mutual exclusivity not as the presence or absence of a mutation but within the evolutionary trajectory of tumor progression [18]. In addition, techniques have been developed that simultaneously infer the exclusivity patterns and recurrent evolutionary trajectories appearing on the tumor’s phylogenetic tree, enabling a more multidimensional understanding of the relationships among mutations in the context of clonal evolution [19,20].

## 3. Within-Tumor Co-Detection: Clonal Architecture and Evolutionary Organization

Most solid tumors comprise genetically diverse subclones generated during tumor evolution. Studies of ITH have broadened the interpretation of mutation co-occurrence from simple co-detection within a bulk sample to analyses within the tumor’s underlying clonal architecture [8,21,22]. In particular, the TRACERx cohort studies, which tracked tumor evolution in non-small cell lung cancer (NSCLC) using multi-region sequencing, demonstrated extensive subclonal diversification. Consequently, mutations co-detected in bulk may include branch-specific events carried by different subclones [23,24].

**Figure 2:**
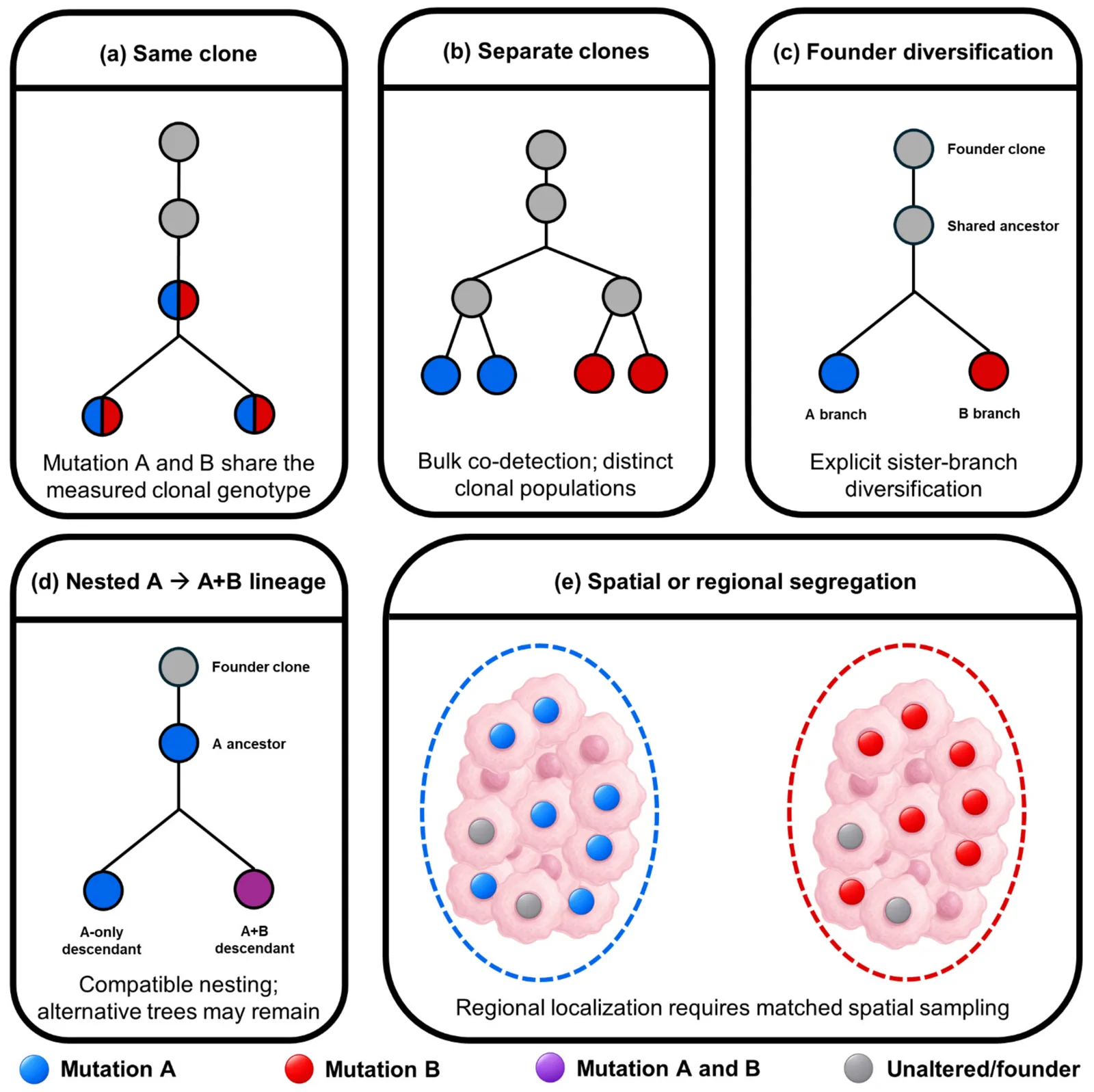
Clonal and spatial configurations underlying within-tumor co-detection: (**a**) Mutations A and B occur within the same measured clone. (**b**) Bulk co-detection arises from separate A- and B-bearing clones. (**c**) A and B occupy sister branches derived from a shared ancestor. (**d**) Mutation B is nested within an A-bearing lineage, although alternative phylogenies may remain compatible. (**e**) A and B are localized to different tumor regions, requiring matched spatial sampling. Blue, red, purple, and gray indicate mutation A, mutation B, A + B, and unaltered/founder populations, respectively. The dashed blue and red ellipses denote distinct spatially sampled tumor regions containing A-bearing and B-bearing cell populations, respectively.

The relationship between two mutations co-detected by bulk sequencing can be considered through several representative clonal configurations, each with distinct evolutionary and functional implications (Figure 2). Their key features, supported interpretations, and principal limitations are summarized in Table 2.

### 3.1. Clonal Configurations Underlying Within-Tumor Co-Detection

The actual relationship between two mutations judged to co-occur within the same tumor through bulk-level analysis must be interpreted by subdividing it into several representative clonal configurations, each of which carries its own evolutionary and functional implications (Figure 2).

Configuration 1: Co-occurrence within the same clone. When mutations A and B are assigned to the same inferred clone, their clonal co-localization is compatible with a cell-autonomous interaction hypothesis, but functional cooperation still requires controlled perturbation (Figure 2a) [22,25].

Configuration 2: Coexistence in different subclones. In this configuration, mutations A and B are detected together in the same tumor sample but reside separately in different subclones along the evolutionary lineage. The resulting bulk-level co-occurrence signal reflects the coexistence within the tumor of cell populations that follow different evolutionary trajectories [8,26]. This distinction is particularly important in tumors with high ITH—such as NSCLC and clear cell renal cell carcinoma—in which multiple subclones compete for proliferative and survival advantage (Figure 2b) [21,22,24]. Experimental studies showed that separation into different subclones does not establish biological independence: paracrine signals, stromal or immune remodeling, metabolic exchange, and shared extracellular resources can support non-cell-autonomous cooperation. Cleary et al. demonstrated cooperation among subclones in Wnt-driven mammary cancers [27]. Marusyk et al. showed that subclonal populations can support tumor growth through non-cell-autonomous interactions [28]. Janiszewska et al. linked subclonal cooperation to metastatic progression through modulation of the local and systemic immune microenvironments [29,30].

Configuration 3: Subclonal diversification from a founder clone. Here, a founder clone bearing a driver alteration diversifies into descendant subclones that acquire different additional alterations. In the NSCLC TRACERx study, alterations in genes including *EGFR*, *MET*, *BRAF*, and *TP53* were classified as clonal in the analyzed tumors, whereas alterations involving *PIK3CA*, *NF1*, and DNA repair-related genes provided examples of subclonal events [24]. These observations illustrate that even mutations detected together in the same tumor occupy different positions within the evolutionary hierarchy, being distributed between the trunk that forms the common ancestral clone and the branches of subsequent subclones. Furthermore, some subclonal mutations may be selectively expanded by the selective pressure exerted during tumor progression and treatment, subsequently becoming markers of the dominant clone [31]. Therefore, even mutations detected together in the same tumor must be interpreted with consideration of the mutational composition and selection process of the subclones branching from the founder clone (Figure 2c).

Configuration 4: Sequential accumulation within the same lineage. Here, an A-positive lineage gives rise to a descendant subclone that subsequently acquires mutation B. That is, all cells carrying mutation B carry mutation A, but not all cells carrying mutation A carry mutation B. In bulk sequencing, CCF or copy-number-corrected VAF differences can make this hierarchy compatible with the data, but CCF_A > CCF_B alone does not prove that B descended from A or that the combination drove a selective sweep. A separate lower-frequency B branch and multiple alternative trees can remain compatible [8,26,32] (Figure 2d).

Configuration 5: Spatial or regional heterogeneity. Finally, two mutations co-detected within the same tumor can be spatially segregated across sampled regions. According to multi-region sequencing studies, even within the same tumor, spatially separated regions can show substantial differences not only in major mutation profiles and CNAs but also in the immune microenvironment; thus, bulk co-detection must likewise be reinterpreted in the context of the three-dimensional spatial heterogeneity of the tumor (Figure 2e) [22,33,34].

### 3.2. Multi-Region and Phylogenetic Analyses

The clonal configurations described above can be investigated by comparing mutation profiles across multiple sampled regions and reconstructing their evolutionary relationships [8,22,24]. Multi-region sequencing helps determine whether mutations detected together in one sample are shared across the sampled regions or are restricted to spatially localized subclones. In a study of patients with clear cell renal cell carcinoma, multi-region sequencing of primary and metastatic tumors demonstrated substantial differences in mutation profiles among sampled regions. Phylogenetic reconstruction supported a branched evolutionary model in which subclones diverge from a founder clone [35]. Likewise, a multi-region sequencing study of NSCLC showed that some mutations corresponded to early clonal events shared across multiple tumor regions, whereas others were confined to specific regions and associated with the formation of descendant subclones [21]. These findings indicate that mutations within the same tumor do not necessarily occupy the same evolutionary tier.

The sampling unit must also be explicit. Cohorts may contain repeated primary, metastatic, relapse, or post-treatment specimens from the same patient; treating them as independent tumors inflates the effective sample size and weights patients unevenly. A cross-sectional patient-level analysis should retain one prespecified specimen per patient or use a model that accounts for within-patient dependence. Lesion-level co-presence and regional co-localization require matched anatomical sampling, and unsampled regions remain a source of uncertainty.

Circulating tumor DNA is a composite analyte. A and B detected in the same plasma sample may originate from one double-positive lesion or from separate metastatic deposits, and lesion-specific shedding varies with site, burden, vascular access, turnover, and treatment [36,37]. Plasma co-detection therefore cannot establish lesion-level, clonal, or same-cell co-occurrence without matched tissue or a source-localization strategy.

Several phylogenetic reconstruction algorithms, including PhyloWGS, CITUP, and ClonEvol, have been developed to analyze bulk and multi-region data. These methods integrate VAF and CNA information derived from bulk sequencing to estimate the CCF associated with each mutation under explicit purity, copy number, multiplicity, and sampling assumptions. Furthermore, they cluster mutations exhibiting similar CCF distributions into common subclonal units and infer parent–child relationships among them, thereby generating one or more phylogenetic reconstructions compatible with the modeled data [26,38,39].

### 3.3. VAF and Clonal Inference

In the analysis of single-sample bulk sequencing data, VAF provides a useful clue for inferring the clonal status of mutations. After adjustment for tumor purity, local copy number, and mutation multiplicity, the CCF of the mutation can be estimated with uncertainty that depends on sequencing depth, purity, local total and allele-specific copy number, mutation multiplicity, and the assumed clonal model. A high CCF indicates high cellular prevalence at the sampled time point; it does not by itself prove early chronological acquisition, and a low CCF does not automatically identify a late event [8,26].

Applying VAF/CCF information can constrain, but not uniquely determine, hierarchical relationships. Two high-CCF alterations may be clonal in the sampled material without having the same acquisition time. If CCF_A exceeds CCF_B, B may be nested within an A-positive lineage or may occupy a separate lower-frequency branch. Pigeonhole, sum, and crossing constraints can reject some configurations, especially across multiple samples, but point estimates should be accompanied by confidence or credible intervals and alternative compatible trees [8,26,32,38,39].

### 3.4. Implications for Therapeutic Targeting

Characterizing the clonal configurations underlying mutation patterns can inform hypotheses for target selection and combination strategies. If two targetable mutations coexist within the same clone and jointly contribute to the survival and proliferation of that clone, combinatorial targeting that simultaneously inhibits both pathways may be effective. Conversely, if the two mutations are segregated into different subclones and each population contributes to disease maintenance, a strategy that addresses both populations may merit evaluation [24,33]. These therapeutic hypotheses remain conditional: same-clone localization does not prove pathway cooperation, while separate clones can cooperate non-cell-autonomously and may require testing of interclonal interactions rather than clone-specific effects alone.

Early trunk alterations inferred to be present in most cancer cells can offer broad target coverage, whereas branch-specific targets may leave genetically distinct populations untreated. Clonality alone, however, does not establish target dependency, therapeutic index, or response. Evolution-informed treatment design should therefore consider both the distribution of a target across sampled clones and the possibility that untargeted populations survive and expand under therapy [22,33].

## 4. Single-Cell Co-Occurrences and Evolutionary Potential

### 4.1. From Clonal Architecture to Cellular Resolution

Multi-region sequencing and phylogenetic inference constrain the clonal composition and evolutionary context of mutation co-detection, but they cannot determine with certainty whether two alterations are present in the same individual cell. These approaches infer cellular prevalence and lineage relationships from population-level VAF and CNA measurements under explicit model assumptions. Same-cell localization therefore requires cell-level or molecule-linked evidence interpreted with appropriate callability and error controls.

In this context, clonal co-occurrence should be distinguished from same-cell co-detection. In general, a clone is defined as a population of cells derived from a common ancestor and sharing a specific combination of mutations. However, even within the same clonal population, ongoing genomic instability can lead to the acquisition of additional mutations or subtle CNA changes, resulting in heterogeneous mutational compositions at the single-cell level [40,41]. Therefore, even when two mutations are assigned to the same clone by bulk or multi-region analysis, their joint distribution at cellular resolution remains to be evaluated with appropriate callability and error controls.

### 4.2. Cellular Configurations of Co-Occurrence

**Figure 3:**
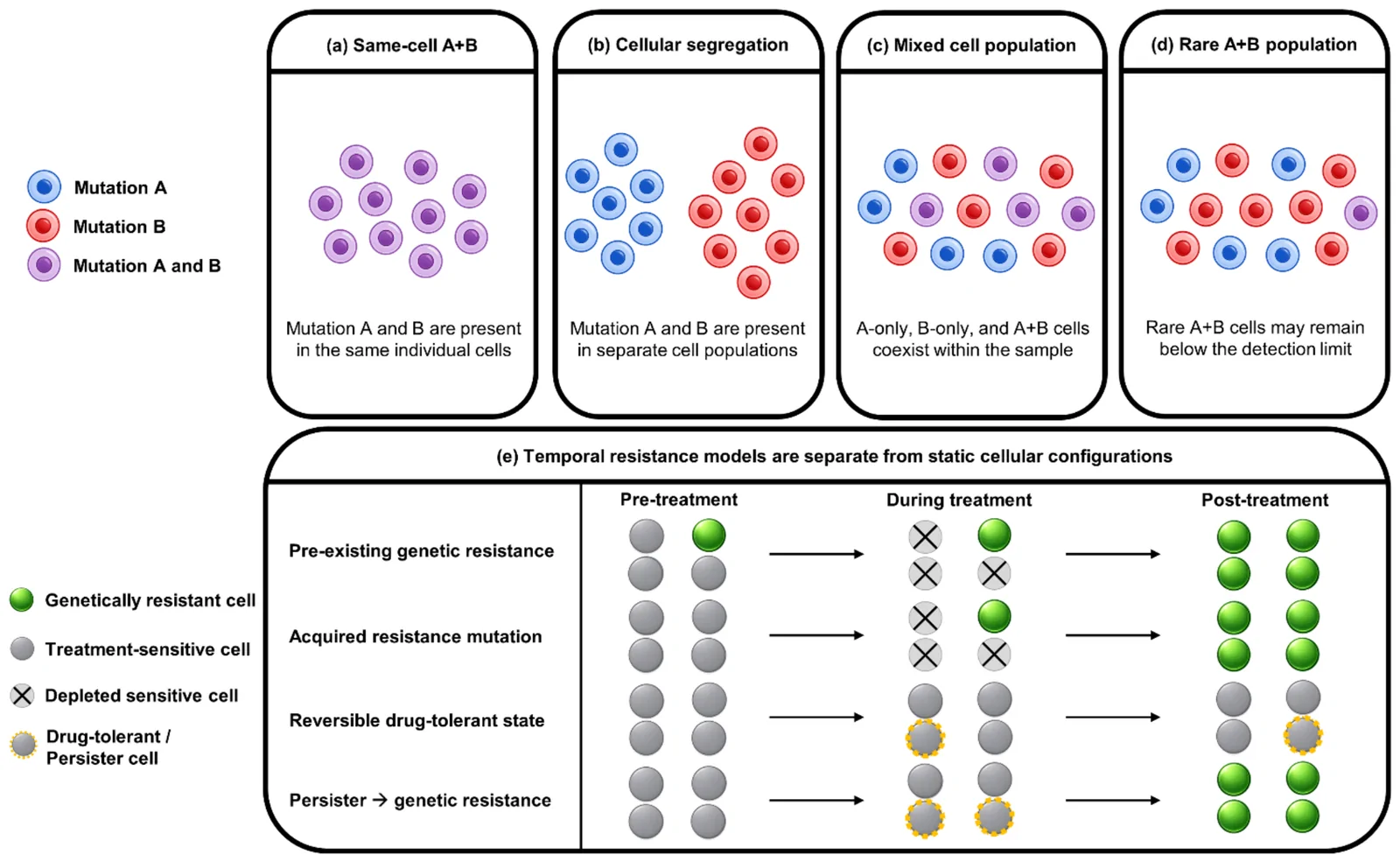
Cellular configurations and distinct routes to treatment resistance: (**a**) Mutations A and B are detected in the same cells. (**b**) A- and B-bearing cells are segregated. (**c**) A-only, B-only, and A + B cells coexist. (**d**) A rare A + B population lies near the detection limit. (**e**) Pre-existing genetic resistance, acquired resistance mutation, reversible drug tolerance, and persister-to-genetic-resistance are shown as distinct temporal models. Green, gray, crossed gray, and yellow-outlined cells denote genetically resistant, sensitive, depleted sensitive, and drug-tolerant persister populations, respectively.

scDNA-seq, which analyzes mutations in individual cells, enables the distribution of multiple mutations detected within the same tumor to be evaluated at the single-cell level. When both loci are callable, these data can distinguish a mixed signal generated by distinct cell populations from joint detection within the same cell or within a low-frequency cellular population. Such cell-level measurements provide higher-resolution evidence for the localization of co-occurrence patterns observed at the clonal level (Figure 3) [41,42,43].

Configuration 1: Co-occurrence within the same cell. This refers to the case in which mutations A and B are simultaneously identified within the same single tumor cell. This finding supports same-cell localization under the stated calling criteria and nominates, but does not prove, a cell-autonomous interaction (Figure 3a) [40,43]. For example, scDNA-seq of patient-derived *TRAF7*/*AKT1*- and *TRAF7*/*KLF4*-mutant meningiomas identified double-mutant cellular genotypes and resolved the order of mutational acquisition [43].

Configuration 2: Segregation across cell populations. Even when mutations A and B are detected together through bulk sequencing, they may not coexist within the same cell at the single-cell level. Instead, they may be segregated into distinct cell populations consisting of mutation A-positive cells and mutation B-positive cells, showing cellular segregation among the sampled, jointly callable cells; non-detection of A + B cells does not establish complete mutual exclusivity without adequate sensitivity and power. Lineage tree analysis based on single-cell mutational profiles is used to infer distinct cellular lineages and branched architectures from these cell-level mutation combinations (Figure 3b) [44]. For example, longitudinal scDNA-seq in a patient with acute myeloid leukemia undergoing IDH2 inhibitor therapy revealed parallel evolution of distinct *IDH1*-, *FLT3*-, *NRAS*-, and *PTPN11*-bearing subclones, illustrating how alterations detected within the same patient can remain segregated across cellular populations [45].

Configuration 3: Mixture of single- and double-mutant cells. Within a tumor, there may coexist cells harboring only mutation A, cells harboring only mutation B, and cells harboring both mutations A and B. For example, single-cell mutation studies in myeloid neoplasms have shown that cell groups carrying different mutation combinations coexist within a single patient sample and that certain subgroups display hierarchical structures characterized by the sequential accumulation of multiple mutations [41]. This indicates that cells with diverse mutational profiles coexist within the same tumor and suggests that cell groups acquiring additional mutations may expand into dominant subclones under subsequent selective pressure (Figure 3c).

Configuration 4: Low-frequency double-mutant populations. Even in environments where cells harboring only a single mutation comprise the majority of tumor cells, a low-abundance cell population carrying both mutations A and B may still exist [41,45]. In one patient with acute myeloid leukemia, scDNA-seq identified *TET2* single-mutant cells together with *TET2*–*U2AF1* double-mutant and more complex descendant populations, providing a direct clinical example of mixed mutation-defined cell populations [45]. Such rare cell populations are difficult to detect because their signals are diluted within the overall tumor signal and fall below the detection limit of bulk sequencing. A recent study of core-binding factor acute myeloid leukemia provides a clinical example of such low-frequency populations: scDNA-seq detected residual tumor cells comprising only 0.16–1.54% of cells during complete remission, including rare cells carrying multiple genomic alterations [40]. A rare pretreatment A + B population should be described as a candidate population unless longitudinal or perturbational evidence links it to subsequent survival and expansion [46]. Strategies for detecting these rare mutant cell populations at the single-cell level and their therapeutic implications are discussed in detail in Section 4.3 and Section 4.4 (Figure 3d).

### 4.3. Detection of Rare Mutant Cell Populations

When the proportion of a cell group carrying a particular mutation is extremely low, the signal from that mutation may be diluted by the major clonal signal of the overall tumor, making detection by bulk sequencing difficult [41,47]. Recent developments include scDNA-seq and joint single-cell DNA/RNA sequencing, which increase the number and types of cell-level measurements available for resolving low-frequency mutation-defined populations [45,48,49]. Increased throughput can improve the probability of detecting rare populations by increasing the number of informative cells, although the effective sensitivity still depends on joint locus callability, allelic dropout, sequencing error, and sampling depth. scDNA-seq analysis can test mutation combinations in individual cells when both loci are callable and technical artifacts are controlled, thereby serving as a useful approach for identifying rare cell populations potentially associated with post-treatment relapse or therapeutic resistance.

Positive and negative single-cell evidence have different error structures. Apparent A + B cells can arise from doublets, contamination, amplification error, or false-positive calls, whereas failure to detect A + B can reflect allelic dropout, incomplete locus coverage, biased capture, or limited cell number [50,51,52]. If the true A + B frequency is f, joint detection sensitivity is s, and n informative cells are sampled independently, detection power is as follows:

(2) $$Power={1-\left(1-fs\right)}^{n}$$

For f = 0.01, s = 0.70, and 95% power, approximately 427 informative cells are required. If zero A + B cells are observed, a rough sensitivity-adjusted 95% upper bound is 3/(ns). The denominator must be the number of cells in which both loci passed prespecified callability criteria, not the nominal number of captured droplets.

scDNA-seq studies conducted in acute myeloid leukemia tracked clonal structures associated with treatment response and relapse at the single-cell level. By detecting mutations at single-cell resolution, these studies reconstructed complex clonal evolution patterns, including linear evolution, branching evolution, and convergent evolution. Furthermore, by integrating longitudinal samples collected from the same patient during treatment, they identified evolutionary trajectories underlying treatment resistance by tracing subclones that survived or expanded into dominant clones under therapeutic selective pressure [45]. Another study performing single-cell mutational profiling in the same cancer cohort demonstrated that clonal distribution patterns may differ according to mutation type. Notably, mutations in epigenetic regulators tended to accumulate together within the same clone, whereas mutations in signaling genes exhibited mutually exclusive patterns by arising independently in separate subclones [41]. Collectively, single-cell analysis can be used to uncover rare subclones that may be obscured by the detection limitations of bulk sequencing and to interpret the mutation combinations they harbor, as well as how they evolve during clonal remodeling before and after therapy. These studies retain platform-, panel-, sampling-, and callability-dependent limitations, so reconstructed lineages and rare-population non-detection should be reported with explicit uncertainty.

### 4.4. Drug Resistance and Non-Genetic Drug Tolerance

Pre-existing genetic resistance, resistance mutations acquired during therapy, reversible drug-tolerant persister states, and post-treatment clonal expansion must be distinguished (Figure 3e). Post-treatment expansion is an observed change in clone abundance and does not by itself identify whether resistance was pre-existing, acquired genetically, or mediated by a reversible cell state. A drug-tolerant state can occur without a stable resistance mutation and may revert after drug withdrawal [53]. Stable genetic resistance can emerge from a pre-existing clone, during therapy, or after a persister state through different evolutionary routes [46,54]. Claims that a pretreatment population predicts resistance require pretreatment ascertainment, a prespecified subsequent outcome, and longitudinal or perturbational validation.

Single-cell multi-omics provides one way to relate genetic resistance and non-genetic cell states without treating them as equivalent. These assays can jointly profile DNA mutations, CNAs, and transcriptional states in the same cell, allowing genotype–state associations to be estimated [48,49]. Integrating these measurements with drug–response data can generate more specific hypotheses; functional and predictive claims still require perturbational and longitudinal validation.

## 5. Methodological Approaches for Resolving Mutational Co-Occurrence and Mutual Exclusivity

A variety of methodological approaches have been developed to detect and interpret mutation co-occurrence and mutual exclusivity, but they address different inferential questions rather than simple successive increasing accuracy. Cohort-level methods estimate statistical dependence, multi-region and phylogenetic methods constrain spatial or clonal placement, and single-cell methods assess cellular co-detection under platform-specific error constraints. Table 1 and Table 3 therefore organize methods by the question, input, assumptions, uncertainty, and failure mode relevant to each analysis.

### 5.1. Cohort-Level Statistical and Combinational Approaches

Cohort-level statistical methods evaluate co-occurrence or mutual exclusivity in binary alteration matrices. Fisher’s exact test provides a basic pairwise test of independence, but it does not account for sample-specific TMB, gene-specific alteration probability, tumor subtype, or cohort composition. Models that incorporate gene- and sample-specific event rates can reduce spurious association or exclusivity signals arising from heterogeneous mutation burdens [3,14,15], whereas models that separate driver and passenger processes address a different source of background rate distortion [16]. Minimum reporting should include the alteration definition; assay coverage/opportunity and the corresponding denominator; A + B/A-only/B-only/neither counts; effect size with a 95% CI; null model; covariates or strata; power considerations; and multiplicity correction. Gene-level binarization can combine functionally distinct variants; panel effects, mutational signatures, same-arm copy number events, and repeated specimens require sensitivity analyses.

Combinatorial optimization-based methods extend beyond individual gene pairs to analyze broader mutation sets composed of multiple genes. Rather than relying on predefined pathways or gene sets, these approaches identify mutually exclusive combinations of genes that are frequently observed across many patients yet rarely overlap within individual tumors, thereby nominating candidate driver modules that may involve shared cancer-related pathways or biological processes [60,61]. In addition, methods combining exact tests conditional on observed alteration frequencies with Markov chain Monte Carlo searches can identify statistically significant mutually exclusive mutation sets that include low-frequency mutations and evaluate whether such patterns recur within particular tumor subtypes [11]. Positive dependencies should be considered alongside exclusivity. SELECT and SelectSim evaluate dependencies from cohort-level alteration patterns, whereas GeneAccord tests whether gene or pathway pairs recur more often than expected in clonally co-occurring or clonally exclusive configurations across patient-specific clone assignments while accounting for evolutionary dependence among clones. These outputs are statistical evidence at their stated resolution and do not establish same-cell localization, functional cooperation, or causality [30,55,56].

### 5.2. Pathway- and Network-Guided Approaches

Pathway- and network-based approaches place mutation relationships within pathways or signaling networks rather than treating them only as pairwise statistical patterns. Specifically, integrating mutations, CNAs, and expression changes with protein interaction networks can nominate connected, mutually exclusive modules [4]. Furthermore, the use of gene sets sharing common downstream signaling effects allows the identification of distinct genetic alterations that converge on similar signaling outcomes. Functional redundancy can generate the hypothesis that co-alteration provides limited additional selective advantage, but a network-supported exclusivity pattern does not by itself prove that mechanism [57]. Moreover, by jointly analyzing patient-level mutation coverage and mutual exclusivity information within protein–protein interaction networks, these approaches can identify combinations of genes that recur across many patients while exhibiting minimal overlap within individual tumors, thereby facilitating the identification of candidate cancer driver modules in the context of network connectivity [58].

### 5.3. Temporal and Evolutionary Approaches

Temporal and evolutionary approaches relate mutation co-occurrence and mutual exclusivity to models of event order during tumor progression. Waiting-time models relate alteration occurrence to the time at which tumors are observed, thereby placing cohort-level exclusivity patterns within an assumed temporal progression model [59].

Pathway-level extensions infer partial orders among pathway events, allowing mutual exclusivity to be interpreted within a modeled progression structure [18]. Furthermore, when major genomic alterations are modeled within tumor progression trees or oncogenetic tree structures, mutation combinations observed across a cohort can be used to infer the relative position of each alteration along a modeled progression trajectory. Through this framework, the observed mutual exclusivity patterns can be interpreted as either events inferred to occur sequentially along a progression pathway or events associated with alternative pathways selected in distinct evolutionary branches [19,20]. These progression models organize cross-sectional patterns under explicit assumptions; they do not directly observe chronological acquisition and should be checked against longitudinal or multi-sample evidence when timing is central.

### 5.4. Clonal and Phylogenetic Inference

Cohort-level mutation patterns can be interpreted within the context of the tumor’s spatial and clonal architecture through multi-region sequencing and phylogenetic clonal inference. Multi-region sequencing analyzes distinct regions of the same tumor to assess whether co-detected mutations are shared across the sampled regions or are restricted to particular regions or subclonal populations. When phylogenetic clonal inference methods are additionally applied, VAF, CNA, and tumor purity information can be integrated to estimate compatible subclonal assignments and evolutionary hierarchies under purity, copy number, multiplicity, and tree assumptions. These approaches can help evaluate whether co-detected mutations are assigned to the same clone, segregated into distinct subclones, or compatible with trunk versus branch placement under the inferred evolutionary model [26,38,39].

### 5.5. Single-Cell and Multi-Omics Approaches

Single-cell analysis provides a higher-resolution method for testing whether mutations co-detected at the bulk level are detected together within the same cell under specified calling criteria. scDNA-seq enables the mutational profile of individual cells to be examined, thereby distinguishing whether two mutations are jointly detected within the same cell, segregated into separate cellular populations, or restricted to low-frequency double-mutant cell populations [41,43,45]. These measurements help determine whether mutation patterns observed as co-occurrence in conventional bulk sequencing represent joint detection at the single-cell level or instead reflect mixed signals arising from distinct clones or cellular populations [41,43].

In addition, single-cell DNA/RNA multi-omics approaches add transcriptional state information. By simultaneously evaluating mutations, copy number status, and RNA-level cellular states within the same cell, these approaches can be used to test whether specific mutation combinations are associated with drug-tolerant states, adaptive states, or therapeutic resistance [48,49]. In particular, these methods can identify candidate genotype–state combinations in low-frequency populations; claims that such populations predict recurrence or resistance require prespecified longitudinal validation [40,41,45,46].

### 5.6. Functional Validation Framework

Statistical association is a discovery signal. Positive association does not establish functional cooperation, same-cell detection does not prove epistasis, clonal segregation does not exclude non-cell-autonomous cooperation, and negative association does not prove synthetic lethality. Claims should be limited to what is supported by the highest completed validation stage. A practical validation sequence is as follows: (1) adjusted discovery with effect size, CI, FDR, and sensitivity analyses; (2) independent replication; (3) lesion, regional, clonal, or cellular localization; (4) an isogenic 2 × 2 comparison of wild type, A, B, and A + B using a prespecified outcome measure; (5) rescue, pathway, competition, co-culture, or mediator blockade studies; and (6) relevant in vivo or longitudinal clinical validation. For cell-autonomous hypotheses, target engagement, dose-response analysis, time-course analysis, and rescue experiments strengthen mechanistic interpretation. For interclonal hypotheses, factorial mixtures of subclones, conditioned medium, transwell or direct co-culture, lineage tracing, and competition experiments can distinguish contact-dependent and secreted-factor effects [27,28,29]. As an example of this validation logic, Wenta et al. tested a context-dependent combined phenotype across engineered cells, organotypic cultures, animal models, and human tissues rather than inferring functional cooperation from a genomic pattern alone [62]. Therapeutic claims require an appropriate null model for the combined effect, assessment of selectivity and toxicity, replication, and context-relevant in vivo evidence. Clinical prediction additionally requires pretreatment ascertainment, a prespecified outcome, temporal precedence, and external or prospective validation.

## 6. Future Directions

Future studies should develop multilevel models that propagate uncertainty from variant calling, copy number estimation, purity, mutation multiplicity, clonal clustering, phylogenetic reconstruction, and single-cell callability into the final association or localization inference. Such models should connect patient, lesion, region, clone, cell, and time while accounting for repeated measurements and alternative compatible clonal configurations. Integration of tissue and circulating tumor DNA will additionally require explicit modeling of unequal lesion shedding and sampling uncertainty. Greater attention is needed to event definition and functional validation. Variant-level and multimodal representations should distinguish hotspot gain-of-function variants, truncating loss-of-function variants, focal copy number events, structural variants, and non-genetic cell states when sample size permits. Cross-cohort analyses should harmonize assay opportunity, panel design, specimen type, treatment context, and event annotation. Artificial intelligence (AI) and machine learning (ML) approaches may complement these analyses by modeling complex genomic backgrounds and prioritizing candidate genetic dependencies. Recent approaches include transfer learning models for element- and cancer-specific background mutation-rate estimation and ML frameworks that translate experimentally measured cancer dependencies to patient tumors to identify patient-relevant vulnerabilities and candidate synthetic-lethal relationships [63,64]. These predictions should be regarded as complementary modeling and prioritization tools rather than direct evidence of biological interaction. They do not by themselves establish physical localization or same-cell residence, and predicted dependencies require independent functional and perturbational validation. Finally, computationally nominated dependencies should be prioritized for perturbational testing using isogenic editing, combinatorial screens, lineage-resolved competition, organoid or co-culture systems, and context-relevant in vivo validation. Together, these advances should improve the interpretation and experimental prioritization of mutation dependencies across spatial and evolutionary contexts.

## 7. Conclusions

The mutation patterns observed in cancer genome data provide important clues for interpreting the relationships among individual mutations; however, their biological significance varies according to multidimensional contexts such as cohort composition, intratumoral clonal architecture, spatial heterogeneity, and therapeutic selective pressures. Interpreting these patterns requires a coherent progression from cohort-level association to within-tumor localization and, where warranted, functional and clinical validation. Claims about within-tumor co-occurrence require localization of bulk co-detection at the lesion, region, clone, or cell level; VAF/CCF and phylogenetic results should be reported with uncertainty and alternative compatible configurations; and the interpretation of single-cell non-detection requires joint callability and power analysis. Mutation co-occurrence is therefore a multilevel signal whose statistical, spatial, functional, therapeutic, and predictive interpretations require distinct but connected forms of evidence.

## Figures and Tables

**Table 1:** Resolution-specific terminology and evidence boundaries.

Unit/Resolution	Preferred Description	Supported Inference	Principal Limitation
Cohort/independent tumors	Positive or negative association	Marginal or covariate-adjusted association	No physical localization or functional inference
Patient	Patient-level co-detection	Both alterations detected across specimens obtained from the same patient	May derive from separate lesions or time points
Lesion	Lesion-level co-presence	Both alterations localized within the same tumor lesion	May occupy different regions or clones
Region	Regional co-localization	Both alterations detected within the same sampled spatial region	Does not establish localization within the same clone or cell
Clone	Clonal co-occurrence/exclusivity	Placement within an inferred lineage or clone	Model and phylogenetic uncertainty; no functional inference
Cell	Single-cell co-detection	Both loci are callable, and both alterations are detected within the same cell	Potential doublets and technical errors; no causal inference
Functional system	Genetic or interclonal interaction	Perturbational effect demonstrated in the tested model	Generalizability requires in vivo or clinical validation

**Table 2:** Comparison of representative clonal and spatial configurations underlying mutation co-detection within tumors.

Configuration	Relationship of A and B	Bulk-Level Pattern	Supported Interpretation	Main Limitation
Co-occurrence within the same clone	A and B are assigned to the same inferred clone	Both show compatible clonal prevalence	Clonal co-localization; compatible with a cell-autonomous interaction hypothesis	Does not prove same-cell residence or functional cooperation
Coexistence in different subclones	A and B reside in distinct subclones	Both are detected in the same tumor	Coexistence of distinct clonal populations	Clonal segregation does not imply biological independence
Subclonal diversification from a founder clone	A founder clone gives rise to descendant subclones with additional alterations	Founder/trunk and descendant/branch alterations are co-detected	Branched evolution from a common lineage	Branching relationships depend on sampling and phylogenetic identifiability
Sequential accumulation within the same lineage	An A-bearing lineage gives rise to an A + B descendant lineage	A has greater cellular prevalence than B	Compatible with sequential accumulation within one lineage	CCF/VAF ordering alone does not prove ancestry
Spatial or regional heterogeneity	A and B are segregated across different tumor regions	Both may be co-detected at the tumor level	Spatial heterogeneity can generate bulk co-detection	Incomplete spatial sampling constrains inference

**Table 3:** Decision guide for selecting statistical and computational methods by inferential target and data structure.

Method/Implementation	Primary Analytical Question	Resolution and Required Input	Heterogeneity or Structure Addressed	Statistical or Model Assessment	Supported Inference	Key Assumptions and Interpretive Limitations
(A) Cohort-level association and dependency analyses						
Fisher’s exact test; CMH; adjusted logistic regression	Is a binary event pair associated before or after stratification or covariate adjustment?	Cohort; binary pair with optional strata or covariates	Observed margins; measured strata, covariates, and interactions	OR, 95% CI, *p* value, and FDR correction	Crude, stratified, or adjusted cohort-level association	Sparse data, residual confounding, non-independent specimens, and no physical localization
DISCOVER [5]	Are two events independent after accounting for heterogeneous event rates?	Cohort; binary tumor-by-event matrix	Gene- and tumor-specific alteration rates	Model-based one-sided *p* values and q values	Pairwise co-occurrence or exclusivity under a heterogeneous-rate null	No direct clinical covariate adjustment or localization; sensitive to event definition
WExT [14]	Is an alteration set more exclusive than expected under weighted mutation probabilities?	Cohort; binary events plus per-sample and per-event probabilities	Sample- and event-specific mutation propensities	Exact or saddlepoint *p* value; multiplicity correction	Statistically significant mutually exclusive event sets	Probability calibration is critical; exclusivity-focused; no localization
MEScan [15]	Which gene sets remain mutually exclusive after background rate adjustment?	Cohort; binary somatic mutation matrix	Patient- and gene-specific background rates; hypermutated genes	Genome-wide testing with multistep FDR control	Mutually exclusive gene sets, including high-confidence sets	Cross-sectional sequence mutation focus; no direct clinical or localization model
CoMEt [11]	Which collections of alteration sets show exact evidence of mutual exclusivity?	Cohort; binary mutation, CNA, or other alteration events	Conditions on event frequencies and searches multiple modules	Exact statistic plus MCMC-sampled solutions	Candidate exclusive collections, including alternative solutions	Sensitive to cohort mixture and event definition; stochastic search dependence
DIALECT [16]	Are latent driver events dependent after separating passenger processes?	Cohort; mutation counts plus a background passenger model	Passenger/background mutation process and latent driver states	Latent-variable parameter estimates and model-based uncertainty	Positive or negative association between inferred driver events	Dependent on the background model; pairwise and cross-sectional; no localization
SELECT [55]	Which curated functional events show positive or negative selection dependencies?	Cohort; curated functional alterations with tumor context	Tumor class and alteration class context	Directional dependency estimates with FDR control	Cohort-level positive or negative functional event dependencies	Depends on event curation; no physical localization or mechanistic proof
SelectSim [56]	Does observed pair incidence differ from TMB-aware simulated independence?	Cohort; oncogenic events, gene rates, and sample TMB	Gene frequencies, sample-level TMB, and cohort partitions	Simulation-based effect size, *p* value, and q value	Positive or negative variant or gene pair dependencies	Limited rare pair power; sensitive to harmonization and TMB definition
(B) Pathway- and network-guided module discovery						
Mutex [57]	Do exclusive alterations converge on a common downstream signaling target?	Network module; alterations plus a directed signaling network	Network constraints and frequency-preserving permutations	Member tests, permutation-calibrated group score, and FDR	Candidate mutually exclusive pathway modules	Depends on network completeness and direction; does not prove functional redundancy
MEXCOwalk [58]	Which connected modules jointly maximize coverage, exclusivity, and connectivity?	Network module; mutation profiles plus a PPI network	PPI topology, alteration coverage, and exclusivity	Random-walk or heuristic module ranking score	Network-supported candidate driver modules	Sensitive to network and tuning choices; not a conventional pairwise association test
(C) Temporal and progression models						
TiMEx [59]	What degree of exclusivity is supported under a waiting-time model?	Progression model; cross-sectional binary events	Alteration waiting times and tumor observation time	Maximum-likelihood estimate and likelihood-ratio test	Temporal exclusivity parameter under the specified model	Strong timing assumptions; cross-sectional data do not directly show order
pathTiMEx [18]	Which mutually exclusive pathways and partial orders explain the data?	Pathway progression; cross-sectional binary events	Pathway membership, within-pathway exclusivity, and order constraints	Generative-model likelihood and stochastic optimization	Mutually exclusive pathways and an inferred partial order	Potential identifiability and scaling limits; timing is model-dependent
ToMExO [20]	Which exclusive driver groups form a tree-structured progression model?	Progression tree; cross-sectional driver events	Tree structure, exclusivity, and observation error	MCMC posterior sampling and model fit	Oncogenetic tree of mutually exclusive driver groups	Depends on tree and event selection assumptions; no spatial or cellular evidence
(D) Clonal and phylogenetic dependency models						
GeneAccord [30]	Are gene or pathway pairs clonally co-occurring or clonally exclusive more often than expected across a cohort?	Clone/tree; patient-specific gene-to-clone assignments, potentially across alternative tree reconstructions	Within-tumor clone structure, evolutionary dependence, and uncertainty across tree assignments	Placement, occurrence, and combined tests; exact or Monte Carlo-calibrated inference as applicable	Cohort-level enrichment of clonal co-occurrence or clonal exclusivity	Depends on clone/tree reconstruction and repeated-cohort occurrence; does not establish same-cell residence, functional cooperation, or causality
TreeMHN [19]	Which dependencies and recurrent trajectories explain a cohort of mutation trees?	Cohort of trees; reconstructed tumor mutation trees	Tree topology, baseline rates, and recurrent evolutionary trajectories	Regularized likelihood or MC-EM; optional stability selection	Dependency network and recurrent trajectory probabilities	Depends on input tree quality, regularization, and sample size; not deterministic prediction

Abbreviations: CI, confidence interval; CMH, Cochran–Mantel–Haenszel; CNA, copy number alteration; FDR, false discovery rate; MC-EM, Monte Carlo expectation–maximization; MCMC, Markov chain Monte Carlo; OR, odds ratio; PPI, protein–protein interaction; TMB, tumor mutational burden.

## Data Availability

No new primary data were generated for the structured narrative-review component of this article. The literature sources are cited in the manuscript, and the complete database search strategies and returned record counts are provided in Appendix A.

## Abbreviations

The following abbreviations are used in this manuscript:

AI	artificial intelligence
CCF	cancer cell fraction
CI	confidence interval
CMH	Cochran–Mantel–Haenszel
CNA	copy number alteration
FDR	false discovery rate
ITH	intratumor heterogeneity
MC-EM	Monte Carlo expectation–maximization
MCMC	Markov chain Monte Carlo
ML	machine learning
NSCLC	non-small cell lung cancer
OR	odds ratio
PPI	protein–protein interaction
scDNA-seq	single-cell DNA sequencing
TCGA	The Cancer Genome Atlas
TMB	tumor mutational burden
VAF	variant allele frequency

## Appendix A. Exact Literature Search Strategy

The following searches were executed on 3 August 2026. Counts are database-returned records and were not treated as screened or included-study counts (Table A1).

**Table A1:** Exact searches and query-return counts.

Database	Search Block	Exact Query	Returned
PubMed/MEDLINE	association_and_ context	(cancer [Title/Abstract] OR neoplasm*[Title/Abstract] OR tumor*[Title/Abstract] OR tumour*[Title/Abstract]) AND (“mutation co-occurrence” [Title/Abstract] OR comutation*[Title/Abstract] OR “co-mutation” [Title/Abstract] OR “mutual exclusivity” [Title/Abstract] OR “genetic interaction” [Title/Abstract] OR epistasis [Title/Abstract]) AND (confound*[Title/Abstract] OR “molecular subtype” [Title/Abstract] OR “Simpson’s paradox” [Title/Abstract] OR aggregation [Title/Abstract])	37
PubMed/MEDLINE	heterogeneity_and_ resolution	(cancer [Title/Abstract] OR neoplasm*[Title/Abstract] OR tumor*[Title/Abstract] OR tumour*[Title/Abstract]) AND (“intratumor heterogeneity” [Title/Abstract] OR “intratumour heterogeneity” [Title/Abstract] OR “cancer cell fraction” [Title/Abstract] OR “variant allele frequency” [Title/Abstract] OR subclone*[Title/Abstract] OR phylogen*[Title/Abstract] OR “multi-region sequencing” [Title/Abstract] OR “multiregion sequencing” [Title/Abstract] OR “single-cell DNA sequencing” [Title/Abstract])	10,338
PubMed/MEDLINE	localization_resistance_ and_liquid_biopsy	(cancer [Title/Abstract] OR neoplasm*[Title/Abstract] OR tumor*[Title/Abstract] OR tumour*[Title/Abstract]) AND (“interclonal cooperation” [Title/Abstract] OR “non-cell-autonomous” [Title/Abstract] OR “circulating tumor DNA” [Title/Abstract] OR “circulating tumour DNA” [Title/Abstract] OR “lesion shedding” [Title/Abstract] OR “drug-tolerant persister” [Title/Abstract] OR “acquired resistance” [Title/Abstract])	18,750
Europe PMC	association_and_ context	TITLE_ABS:(cancer OR neoplasm* OR tumor* OR tumour*) AND TITLE_ABS:(“mutation co-occurrence” OR comutation* OR “co-mutation” OR “mutual exclusivity” OR “genetic interaction” OR epistasis) AND TITLE_ABS:(confound* OR “molecular subtype” OR “Simpson’s paradox” OR aggregation)	35
Europe PMC	heterogeneity_and_ resolution	TITLE_ABS:(cancer OR neoplasm* OR tumor* OR tumour*) AND TITLE_ABS:(“intratumor heterogeneity” OR “intratumour heterogeneity” OR “cancer cell fraction” OR “variant allele frequency” OR subclone* OR phylogen* OR “multi-region sequencing” OR “multiregion sequencing” OR “single-cell DNA sequencing”)	10,932
Europe PMC	localization_resistance_ and_liquid_biopsy	TITLE_ABS:(cancer OR neoplasm* OR tumor* OR tumour*) AND TITLE_ABS:(“interclonal cooperation” OR “non-cell-autonomous” OR “circulating tumor DNA” OR “circulating tumour DNA” OR “lesion shedding” OR “drug-tolerant persister” OR “acquired resistance”)	18,959

* The asterisk denotes a truncation wildcard used to retrieve all terms beginning with the specified word stem (e.g., tumor retrieves tumor and tumors).
